# Recovery of Bioactive Compounds from Pomegranate Seeds (*Punica granatum* L.) Using Microwave- and Ultrasound-Assisted Extraction Techniques

**DOI:** 10.3390/plants15081247

**Published:** 2026-04-18

**Authors:** Wendy Magaly Arias-Balderas, Elba Ronquillo-de Jesús, Omar Patiño-Rodríguez, Chelsi Amairani Cortes-Reyna, Miguel Angel Aguilar-Méndez

**Affiliations:** 1 Secretaría de Ciencia, Humanidades, Tecnología e Innovación (SECIHTI), Ciudad de Mexico 03940, Mexico; wen.ariasb@gmail.com; 2 Centro de Investigación en Ciencia Aplicada y Tecnología Avanzada (CICATA)-Legaria, Instituto Politécnico Nacional, Ciudad de Mexico 11500, Mexico; 3 Dirección de Ingeniería Agroindustrial, Universidad Politécnica de Francisco I Madero, Tepatepec 42660, Hidalgo, Mexico; eronquillo@upfim.edu.mx (E.R.-d.J.); chelsi.amairani@gmail.com (C.A.C.-R.); 4 SECIHTI-CEPROBI, Instituto Politécnico Nacional, Yautepec 62739, Morelos, Mexico; opatinor@ipn.mx

**Keywords:** pomegranate by-product, phenolic compounds, antioxidant activity, microwaves, sonication

## Abstract

In this study, we compared the effects of microwave-assisted extraction (MAE) and ultrasound-assisted extraction (UAE) on the total phenolic content, antioxidant activity, morphological characteristics, and tentative identification of bioactive compounds by LC-ESI-MS/MS in pomegranate seeds. We conducted a phytochemical characterization of the extracts by determining the total phenolic content and total flavonoids. Antioxidant activity was evaluated using ferric-reducing antioxidant power (FRAP) and free radical inhibition methods (DPPH and ABTS). Morphological characteristics were analyzed via scanning electron microscopy, UV-Vis and FTIR of the extracts were recorded. Additionally, the main bioactive compounds were identified using HPLC-MS. Our results demonstrated that MAE was the most efficient technique, yielding a higher content of total phenols (35.47 mg GAE/g), total flavonoids (14.44 mg CAE/g) and antioxidant activity (0.19 and 0.41 mmol TEAC/g, as determined by FRAP and ABTS, respectively). In terms of morphological characteristics, UAE induced more changes in the structure of the plant material compared to MAE. According to HPLC-MS analysis, the extract obtained using MAE notably contained coumaric acid, cyanidin, and quercetin, whereas the UAE extract included coumaric acid, cyanidin, kaempferol, and epicatechin. In conclusion, this study demonstrated that MAE is a more efficient method than UAE for extracting bioactive compounds. Pomegranate seeds may represent a potential source of these compounds for application in various industrial areas.

## 1. Introduction

The pomegranate (*Punica granatum* L.), a plant from the *Punicaceae* family, is native to the Mediterranean region. Due to its adaptive nature, it is now cultivated in tropical and subtropical areas globally [1]. In Mexico, cultivation spans 18 states, with Morelos, Hidalgo, and Oaxaca being the primary producers. According to the Ministry of Agriculture and Rural Development (SADER) [2], just over 10,000 tons were produced in 2022.

Juice, the primary product derived from pomegranate fruit processing, constitutes roughly 30% of the fruit’s total weight. This processing, however, results in significant waste, as peels and seeds account for about 50% of the fruit’s weight [3]. Pomegranate fruit residues are rich in phytonutrients, which offer substantial health benefits. Notably, the seeds contain approximately 1.2% polyphenols, including anthocyanins, ellagic acid, and kaempferol [4].

Phenolic compounds are a significant class of phytochemicals found in plants, comprising a wide array of derivatives such as simple phenols, phenylpropanoids, benzoic acid derivatives, flavonoids, tannins, lignans, and lignins. These compounds have gained noteworthy scientific and industrial attention due to their reported benefits in various studies, including antioxidant capacity, as well as antimicrobial, antifungal, anti-inflammatory, antidiabetic, and anticancer properties, among others [5]. Consequently, there is an urgent need to optimize extraction methods to enhance both the yield and the biological activity of these bioactive compounds.

The extraction process is a crucial step that determines both the yield and bioactive properties of secondary metabolites, such as polyphenols. Traditional methods typically require high temperatures and extended periods, leading to substantial energy consumption. These conditions can be too harsh for heat-sensitive compounds. Additionally, conventional methods necessitate large volumes of organic solvents [6]; to mitigate these drawbacks, emerging extraction technologies are being explored. These innovative methods optimize the extraction process and yield improved results due to their distinct mechanisms of action. Notably, microwave-assisted extraction (MAE) and ultrasound-assisted extraction (UAE) are among the most promising emerging methods [7,8,9].

MAE has garnered significant attention due to its unique heating mechanism, moderate capital cost, and effective performance under atmospheric conditions. Microwaves, consisting of an electric field and a magnetic field oscillating perpendicularly, operate at frequencies between 0.3 and 300 GHz. They can penetrate certain materials and interact with polar components to generate heat through ionic conduction and dipolar rotation. This selective heating depends on the material’s dielectric constant [10].

Conversely, the UAE is a viable alternative for extracting natural compounds. This technique employs low solvent volumes and short extraction times, resulting in higher yields and minimizing the degradation of thermosensitive compounds [11]. Sonication involves using high-intensity sound waves, which cause physical alterations in plant tissues through acoustic cavitation. This process enhances the release of extractable components into the solvent by improving mass transfer [12].

Although a significant amount of research exists on obtaining bioactive compounds from pomegranate fruit, these studies primarily focus on the use of the peel and pulp. Conversely, reports on the use of the seed as a source of bioactive compounds are scarce. Furthermore, the reported studies mainly employ conventional extraction techniques, such as maceration. In this regard, the present research stands out for its use of non-conventional techniques, such as UAE and MAE. Moreover, the results obtained help strengthen our understanding of the effect that each mechanism produces on the plant matrix and the types of compounds obtained. Therefore, the objective of this study was to compare the effects of two non-conventional extraction processes—MAE and UAE—on total phenolic content, total flavonoids, and antioxidant activity (measured by DPPH, FRAP, and ABTS assays). Additionally, it provided information on the bioactive compounds present in both extracts using HPLC-MS.

## 2. Results and Discussion

### 2.1. UV-Vis and Infrared Spectroscopies

**Figure 1:**
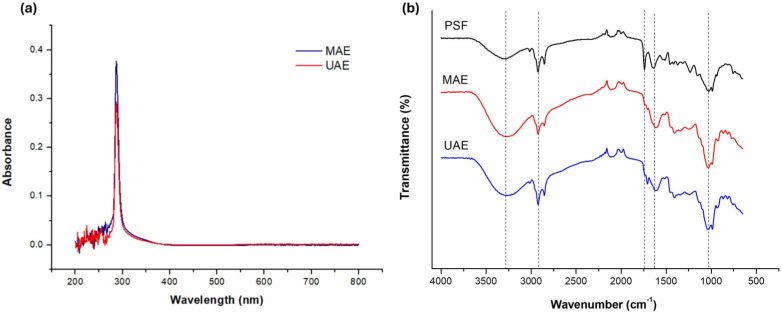
(**a**) UV-Vis and (**b**) FTIR spectra for extracts obtained by MAE and UAE.

Figure 1 presents the UV-Vis and FTIR spectra of extracts obtained via MAE and UAE methods. The UV-Vis spectra (Figure 1a) of both extracts display a predominant band in the range of 280–300 nm, with a peak absorbance at 287 nm. As noted by Catalin Mot et al. [13], this region of the UV-Vis spectrum is indicative of phenolic compounds. Flavonoids, a subgroup of these compounds, typically exhibit absorption bands between 240–280 nm and 300–400 nm [14]. In pomegranate seeds, commonly reported flavonoids include kaempferol and anthocyanins, with (+)-catechin and (-)-epicatechin also likely present, which have peak absorbances at 280 and 278 nm, respectively. While the spectra are largely similar, the MAE extract demonstrates higher absorbance, suggesting a greater concentration of phenolic compounds.

FTIR analysis is effective for determining major functional groups and identifying potential changes in a sample’s chemical composition. The FTIR spectra of pomegranate seed flour (PSF) and the extracts obtained through EAM and EAU are illustrated in Figure 1b. A strong and broad signal was consistently recorded in the 3200–3350 cm^−1^ region, indicating the stretching vibrations of -OH groups, with the processed samples displaying the greatest intensity. The band at 2900 cm^−1^ corresponds to the stretching vibrations of -CH bonds found in the aromatic methoxy groups of carboxylic acids, as well as in the alkyl chains [15]. According to Rashid et al. [16], the peak at 1750 cm^−1^ is associated with the stretching vibrations of C=O bonds, characteristic of carbonyl or ester groups. It is noteworthy that this signal appeared well-defined in the seed spectrum; however, its intensity significantly decreased in the spectrum of the UAE extract and disappeared in the one obtained by MAE. This change occurs because the band is related to aldehyde or ketone groups primarily found in carbohydrates (though they may also be present in other seed matrix components), which are preferentially removed during the centrifugation and filtration process. The signal at 1605 cm^−1^, intensified in the spectra of both extracts, corresponds to the stretching vibrations of the C=C bond in the benzene ring or amides [3]. Finally, an intense and broad band observed in the 1000–1086 cm^−1^ region is attributed to the stretching vibrations of C-O bonds in carboxylic acids and C-OH in alcohol groups, characteristic of phenolic compounds [17]. In conclusion, FTIR analysis effectively recorded the main functional groups indicative of phenolic compounds in the extracts, and no changes in chemical composition resulting from the extraction processes were identified.

### 2.2. Total Phenolic Content and Flavonoid Content

**Figure 2:**
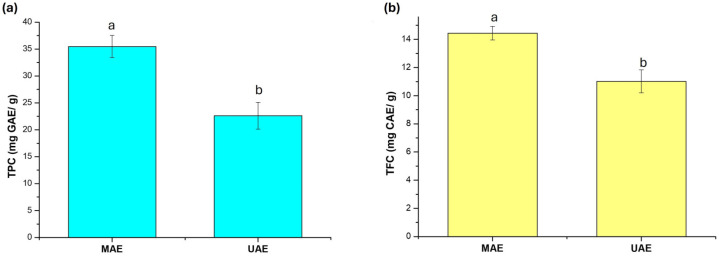
Effect of the extraction method on (**a**) total phenolic content and (**b**) total flavonoid content in pomegranate seed extracts. Different letters in the bars show significant difference by LSD test (*p* ˂ 0.05).

To evaluate the effect of the extraction method on the yield of phenolic compounds in pomegranate seed extract, a comparative study was conducted between MAE and UAE (Figure 2). Figure 2a indicates that the total phenolic content was higher in seed extracts obtained via MAE (35.47 mg GAE/g of dry extract), with this value being significantly different (*p* < 0.05) from that recorded for UAE (22.62 mg GAE/g of dry extract). These values exceed those reported by Zhang et al. [4], who discovered total phenolic contents between 9.94–10.36 mg/g in pomegranate seed extracts processed using eutectic solvents and UAE. For total flavonoids, the trend was similar; MAE produced a statistically higher amount (*p* < 0.05) of total flavonoids (14.44 mg CAE/g of dry extract) compared to UAE (11.02 mg CAE/g of dry extract) (Figure 2b). This outcome can be attributed to the findings of Cheng et al. [18], which suggest that MAE’s effectiveness arises from enhanced desorption, diffusion, and dissolution processes of bioactive compounds. The microwave incidence increases internal cell pressure, alters the physical properties of biological tissues, and improves the porosity of the biological matrix. This enhancement allows better solvent penetration and increases the yield of desired substances. Nayak et al. [19] reported similar results when comparing MAE and UAE regarding the total phenolic content of orange peel extracts.

It should be noted that differences observed between MAE and UAE extracts may be influenced not only by the extraction technique itself, but also by the operational parameters applied (time and temperature), which were not standardized in this study.

### 2.3. Antioxidant Capacity

The antioxidant capacity of an extract is closely linked to the ability of phenolic compounds to scavenge free radicals, disrupt radical chain reactions, and chelate metals [19]. Common methods for determining antioxidant activity focus on free radical inhibition or neutralization reactions, such as DPPH and ABTS, or metal ion reduction, as seen in the FRAP assay [20].

Table 1 presents the values obtained from the three tests conducted. The FRAP results indicate that both extraction methods yielded extracts capable of reducing Fe^3+^ ions to Fe^2+^ ions. However, the extract from the MAE method recorded the highest Trolox equivalent value (0.19 mmoles TEAC/g), compared to the extract from the UAE method (0.13 mmoles TEAC/g), which was a statistically significant difference. Additionally, these values exceeded those reported by Kalaycıoğlu and Erim [21] for seeds of six pomegranate ecotypes from Tunisia, which ranged from 0.053 to 0.1 mmol/g.

The DPPH assay indicated that the MAE method resulted in a slightly higher value than the UAE method. This implies that a greater quantity of microwave extract is necessary compared to the ultrasound extract to inhibit the radical by 50%. Statistically, there was a significant difference between the two extraction methods. Despite this, the IC_50_ values for both methods were small, demonstrating that only a minimal amount of extract is needed to inhibit 50% of the DPPH radical. This finding translates into high antioxidant activity [22].

The results from the ABTS method indicated that the values for both the MAE and UAE methods were higher than those from the other methods (FRAP and DPPH), measuring 0.41 and 0.39 mmol TEAC/g, respectively. This outcome was anticipated, as higher values indicate a superior ability to inhibit free radicals, underscoring the significant antioxidant capacity of pomegranate seeds. The value obtained by UAE was slightly lower than that of MAE, with the difference being statistically significant (*p* < 0.05). Similar findings were reported by Pourshoaib et al. [23], who observed comparable trends using the DPPH and ABTS methods on date seeds. They also found that UAE yielded slightly lower values than MAE, with a statistically significant difference.

Under the conditions utilized in this study, the MAE method yielded superior results, demonstrating a statistically significant difference. Moreover, it was shown to achieve a greater antioxidant effect in half the time compared to UAE. This effectiveness may be attributed to the MAE method’s reliance on electromagnetic waves, which can directly target polar substances due to their high dielectric constant values. Consequently, this facilitates the extraction of polyphenols—hydrophilic compounds largely responsible for the antioxidant capacity of plant materials [12].

### 2.4. SEM Microscopy

**Figure 3:**
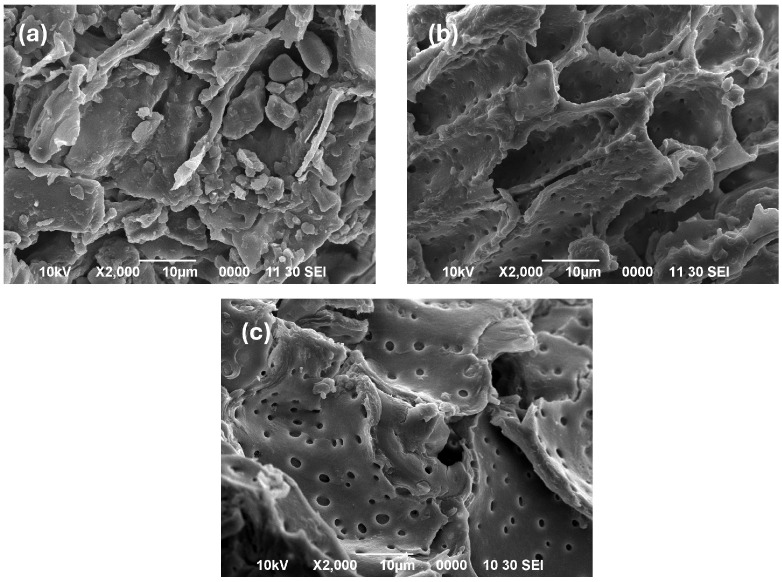
Micrographs obtained by SEM of the flour and residues. (**a**) Untreated pomegranate seed, (**b**) residue obtained by MAE and (**c**) residue obtained by UAE.

SEM analysis was employed to examine the morphology and microstructural characteristics of the sample surfaces. Figure 3 displays the SEM micrographs of pomegranate seed flour and residues following extraction by MAE and UAE methods. In the raw sample image (Figure 3a), a densely packed surface with a relatively intact plant wall is evident, showing only slight mechanical damage from grinding. Additionally, basic structures such as starch granules are present, and no porosities are observed. In contrast, the extraction processes significantly altered the physical characteristics of the plant material. Figure 3b reveals that microwave application almost completely removed the material within the cellular structures and caused apparent damage to the cell wall’s outer layers. Nevertheless, despite the conditions generated by electromagnetic energy, the internal structure was somewhat preserved. According to More and Arya [24], the effectiveness of microwave irradiation in breaking the cell wall is attributed to ionic conduction and the permanent dipolar rotation of molecules, which results in rapid solvent heating. The structural changes allow the solvent to easily infiltrate cellular matrices, enhancing the extraction efficiency. Furthermore, the sample is heated uniformly, and the heat generated reaches the solid material directly, without being absorbed by the solvent. Unlike the observations in Figure 3b, UAE induced more pronounced morphological changes in the plant material structure (Figure 3c), evidenced by the complete collapse of the cellular structure. Fragmented structures and large cracks reflect the cavitation generated during the sonication process. This phenomenon of cavitation causes the membranes to rupture by inducing physical and mechanical changes, thereby facilitating the penetration of the solvent and its contact with the structures inside the cell [25]. Although the application of ultrasonic waves facilitates the diffusion and release of polyphenols within an appropriate time frame, prolonged exposure leads to the decomposition of certain polyphenols due to increased thermal and mechanical effects [26].

Although this study did not consider the effect of extraction time, the duration for UAE was twice that of MAE, which might explain the lower total phenolic content in extracts obtained by UAE.

### 2.5. HPLC-ESI-MS Compound Identification

**Figure 4:**
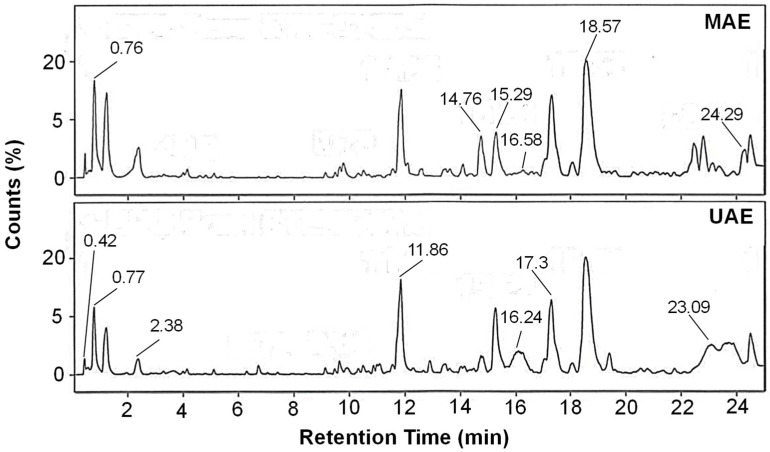
Base peak chromatograms (BPC) of pomegranate seed extracts. Peaks were tentatively assigned based on LC-ESI-MS/MS analysis, including accurate mass, fragmentation patterns, and database matching; therefore, compound identification should be considered putative.

**Figure 5:**
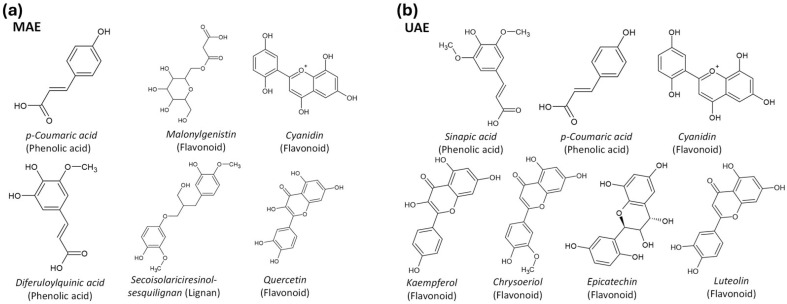
Structures correspond to the most representative isomeric forms based on LC-MS annotation and database matching. Compounds extracted by (**a**) MAE and (**b**) UAE.

An analysis of pomegranate seed extracts, recognized for their high phenolic content, was conducted using LC-ESI-MS/MS to identify a variety of present compounds. The focus was on detecting compounds associated with antioxidant activity, providing insights into the phenolic profile of the extracts, and highlighting several key compounds with such activity; the resulting chromatogram is shown in Figure 4. Importantly, the chemical composition of an extract depends on the extraction method utilized. For instance, notable compounds identified in the extract obtained by MAE included coumaric acid, cyanidin, and quercetin (Table 2 and Figure 5a). In contrast, the extract obtained by UAE contained coumaric acid, cyanidin, kaempferol, and epicatechin (Table 3 and Figure 5b). These differences in constituents are directly linked to the predominant extraction mechanisms of each technique. The findings in this study align with previous research, confirming the reputation of pomegranate seed extract as a rich source of bioactive molecules [27]. Each phenolic compound plays a significant role in antioxidant defense mechanisms. Specifically, quercetin and kaempferol are known for their anti-inflammatory and anticancer properties [28,29], while epicatechin is associated with cardiovascular health benefits [30]. The diversity of phenolic compounds detected suggests that pomegranate seed extracts may have potential applications in health, including the development of nutraceuticals and functional foods.

Compounds were identified by comparing the experimental *m*/*z* values and MS/MS fragmentation patterns with spectral data available in the mzCloud and MassBank databases. For clarity, glycosylated or derivatized compounds were simplified to their corresponding aglycone forms. Theoretical molecular weights were obtained from these databases and were further cross-validated using public chemical repositories such as PubChem and previously reported literature when available. Identification confidence was supported by accurate mass error (<5 ppm), isotopic distribution, and fragmentation matching. The LC-ESI-MS chromatogram (Figure 4) shows the separation profile of the extract, where the peaks corresponding to the identified phenolic compounds are indicated. Compound identification was based on accurate mass measurements, isotopic distribution, MS/MS fragmentation patterns, and database matching (mzCloud, MassBank, and PubChem). Therefore, all compounds should be considered as putatively annotated, and their identification remains tentative due to the absence of authentic standards. For clarity, glycosylated or derivatized compounds were simplified to their corresponding aglycone forms when reporting the main identified compounds. Therefore, all compound identifications should be considered tentative due to the absence of authentic standards.

## 3. Materials and Methods

### 3.1. Materials

The following reagents were used in the study: Folin-Ciocalteu reagents, 2,4,6-tripyridyl-s-triazine (TPTZ), 2,2-azinobis(3-ethyl-benzothiazoline-6-sulfonic acid) diammonium salt (ABTS), and 2,2-diphenyl-1-picrylhydrazyl (DPPH), all of which were purchased from Sigma-Aldrich (St. Louis, MO, USA). Sodium carbonate was obtained from Merck (Darmstadt, Germany), while iron (III) chloride hexahydrate was sourced from Deiman (Mexico City, Mexico). Technical-grade ethanol, acquired from Meyer (Mexico City, Mexico), was also used in the experiments.

### 3.2. Conditioning of Raw Materials

Mollar pomegranate fruits at commercial maturity were harvested between July and August 2023, in a farm located in the Chilcuautla area, in the state of Hidalgo, Mexico. (20°14′ and 20°25′ north latitude, 99°09′ and 99°22′ west longitude). This geographical area is characterized by a predominantly temperate semi-arid climate. Fruits were washed with running water and sanitized with a 1% sodium hypochlorite solution for 10 min. Seeds were then removed and dehydrated at 55 °C for 24 h in a food dehydrator, until they reached a moisture content of 9.4%. This value was determined using a thermobalance (MB 120, Ohaus Corporation, Parsippany, NJ, USA) at 105 °C for 10 min.

The dried material was ground with a hammer mill (TS3383L60, Thomas Scientific, Swedesboro, NJ, USA) and sieved through a #40 mesh stainless steel sieve (0.420 mm).

### 3.3. Microwave-Assisted Extraction

A 12.5 g plant sample was incorporated into 250 mL of a 70:30 ethanol–water solution through manual stirring. The mixture underwent microwave irradiation using a digestion system (LMWD-A10, Labtron, Camberley, UK) at 1600 W for 15 min at 65 °C. After irradiation, the mixture was decanted and centrifuged at 515× *g* (K 3454OM, International centrifuge, Philadelphia, PA, USA). The supernatant was collected, and the sediment underwent a second extraction under identical conditions. The resulting supernatants were combined, filtered, and concentrated using a rotary evaporator (RE500, Yamato Scientific Co., Tokyo, Japan) at 40 °C. Finally, the extract was lyophilized (FreeZone 4.5, Labconco, Kansas City, MO, USA) and stored at room temperature in the dark.

### 3.4. Ultrasound-Assisted Extraction

A mixture containing 12.5 g of plant material and 250 mL of an ethanol–water solution (70:30) was sonicated at 25 kHz for 30 min in an ultrasonic bath (TI-H-5, Elma, Singen, Germany) at an initial temperature of 25 °C. The final temperature was recorded after 30 min, which was 50 °C. Following sonication, the mixture was decanted and centrifuged at 515× *g* (K 3454OM, International centrifuge, Philadelphia, PA, USA). The supernatant was collected, and the sediment underwent a second extraction under identical conditions. The supernatants from both extractions were combined, filtered, and concentrated using a rotary evaporator (RE500, Yamato Scientific Co., Tokyo, Japan) at 40 °C. Finally, the extract was lyophilized using a FreeZone 4.5 (Labconco, Kansas City, MO, USA) and stored at room temperature in the dark.

### 3.5. Total Phenol Content

The total phenol content was determined using the Folin–Ciocalteu assay, based on the methodology described by Villamil-Galindo and Piagentini [31], with some modifications. In a 96-well microplate, 25 µL of diluted extract was placed in each well. Subsequently, 125 µL of distilled water, 20 µL of Folin’s reagent (diluted tenfold 1:10), and 30 µL of sodium carbonate were added. After allowing the mixture to rest for 30 min in the dark, absorbance was measured at 760 nm using a spectrophotometer (Multiskan GO, Thermo Fisher, Vantaa, Finland). Gallic acid served as the standard. Meanwhile, a mixture of the reagents + 150 µL of distilled water, served as the blank. The results are expressed as mg of gallic acid equivalent (GAE)/g of dry extract.

### 3.6. Total Flavonoid Content

The procedure was adapted from the methodology of Farooq et al. [32] with some modifications. A 0.5 mL sample of diluted extract was combined with 2.5 mL of distilled water + 0.15 mL of 5% NaNO_2_ in a 15 mL Falcon tube. The mixture was allowed to stand for 6 min. Subsequently, 0.3 mL of 10% AlCl_3_·6H_2_O was added, and after a further standing period of 5 min, 1 mL of 5% NaOH was added. The solution was then vortexed vigorously for 1 min and the absorbance was immediately measured at 510 nm. Catechin served as the standard, and results are expressed as mg catechin equivalents (CAE)/g of dry extract.

### 3.7. FRAP

The iron reduction antioxidant power (FRAP) was assessed according to the method described by Gullón et al. [33], with some modifications. In each well of a 96-well microplate, 20 µL of diluted extract was combined with 180 µL of FRAP solution and 60 µL of distilled water. This mixture was incubated at 37 °C for 10 min under dark conditions, after which the absorbance was measured at 593 nm. Trolox was used as a standard, and the results are reported as micromoles of Trolox equivalent (TEAC)/g of dry extract.

### 3.8. DPPH and ABTS

The free radical inhibition activity was assessed using the DPPH method [34], with certain adjustments. In a well of a 96-well microplate, 200 µL of diluted extract was combined with 50 µL of DPPH stock solution. This mixture was allowed to stand in the dark for 30 min. Absorbance was measured at a wavelength of 515 nm. Results are expressed as micromoles of Trolox equivalent (TEAC)/g of dry extract. Furthermore, the concentration required to inhibit 50% of DPPH (IC50) was determined by plotting the % degraded DPPH VS concentration of the extract.

The antioxidant capacity using the ABTS method was assessed following the methodology outlined by Rumpf et al. [35], with some modifications. In a well of a 96-well microplate, 25 µL of diluted extract was combined with 180 µL of ABTS solution. The mixture was left to stand for 10 min under dark conditions, after which the absorbance was measured at a wavelength of 734 nm. Trolox served as the standard, and results are expressed as micromoles of Trolox equivalent (TEAC)/g of sample.

### 3.9. Scanning Electron Microscopy (SEM)

Pomegranate seed powders’ morphology and physical characteristics, both before and after extraction, were examined using an electron microscope (JMS-6390LV, JEOL, Tokyo, Japan). Samples were coated with gold for 120 s using a sputter system (Desk IV, Denton Vacuum, Moorestown, NJ, USA) to prepare them for observation. Micrographs were then obtained at various magnifications.

### 3.10. UV-Vis and Fourier Transform Infrared (FTIR) Spectroscopy

Scanning was conducted over a wavelength range of 200–800 nm using a Genesys spectrophotometer (10S, Thermo Scientific, Waltham, MA, USA). Infrared analysis aimed to identify the different functional groups present in the extracts obtained by both methods: MAE and UAE. A Cary 630 device (Agilent Technologies, Santa Clara, CA, USA) facilitated this analysis. Signals were obtained in the region of 4000–500 cm^−1^. The analysis utilized an attenuated total reflectance cell with a resolution of 4 cm^−1^ across 32 scans.

### 3.11. HPLC-ESI-MS Analysis

The extracts obtained by both methods were analyzed using HPLC with an Agilent 1260 system. This system was equipped with a quadrupole pump, degasser, and diode array detectors, and was coupled to an Agilent 6530 mass spectrometer (Agilent Technologies, Santa Clara, CA, USA) featuring electrospray ionization and a quadrupole time-of-flight (ESI-QToF) detector. For each sample analysis, a Poroshell 120 EC-C18 column (3.0 mm × 50 mm × 2.7 µm) was used at 30 °C. The mobile phase consisted of 0.1% formic acid in water (eluent A) and acetonitrile (eluent B). A gradient elution method was employed, starting with initial conditions of 95% A and 5% B, transitioning to 80% A at 0–5 min, 10% A at 5–20 min, and finally 0% A at 20–24 min. The total run time was 24 min, with a flow rate of 0.5 mL/min and an injection volume of 2.5 µL. Mass analysis was performed in the positive ionization mode, with a gas temperature of 275 °C and a flow rate of 7 L/min. The capillary voltage was set at 4500 V, and the nebulizer pressure at 30 psi. The resulting chromatograms were analyzed by matching the experimental spectra with the mzCloud and MassBank databases. Compound identification was based on accurate mass, isotopic pattern, and MS/MS fragmentation matching. Theoretical molecular weights were obtained from these databases and corroborated using public repositories (PubChem) and literature reports.

### 3.12. Statistical Analysis

All analyses were conducted in triplicate, and results are expressed as mean ± standard deviation. We used OriginPro 9.0 software to perform an analysis of variance (ANOVA). Additionally, a comparison of means was carried out using the Least Significant Difference (LSD) test at a significance level of *p* < 0.05.

## 4. Conclusions

This study compared the efficiency of MAE and UAE in extracting bioactive compounds and assessing the antioxidant activity of pomegranate seed extracts. The results demonstrated that, under the conditions used in this study, MAE was superior to UAE, yielding a higher concentration of phenolic compounds and extracts with enhanced antioxidant activity. The total phenolic content showed a positive correlation with antioxidant activity, as measured by FRAP, ABTS, and DPPH assays. The HPLC-MS analysis revealed a diverse array of phytochemicals, including flavonoids and phenolic compounds, with profiles varying based on the extraction technique employed. This diversity suggests that pomegranate seeds are a valuable source of antioxidants, offering potential applications in the food and pharmaceutical industries. Additionally, utilizing agro-industrial waste supports the circular economy and mitigates environmental pollution from waste. Furthermore, the results obtained provide a broader perspective on the use of these extraction techniques in future studies. Among these, the use of the same temperature and time conditions for both methods was evaluated. The optimization of extraction processes using response surface methodology was also considered. Finally, it is suggested that the presence of tannins be determined, since, according to the literature, these compounds are present in pomegranate fruit. However, compound identification remains putative, and further studies using authentic standards and standardized extraction conditions are required to confirm compound structures and enable a more controlled comparison between extraction techniques.

## Figures and Tables

**Table 1:** Antioxidant capacity of the extracts obtained by MAE and UAE.

Method	FRAP (mmol TEAC/g)	DPPH IC_50_ (mmol TEAC/g)	ABTS (mmol TEAC/g)
MAE	0.19 ± 0.03 ^a^	0.17 ± 0.01 ^a^	0.41 ± 0.02 ^a^
UAE	0.13 ± 0.00 ^b^	0.15 ± 0.00 ^b^	0.39 ± 0.07 ^b^

Different letters in the same column show significant difference by LSD test (*p* ˂ 0.05). Values expressed as mean ± standard deviation. All analyses are expressed as micromoles of trolox equivalent (TEAC).

**Table 2:** Putative compound identification of extracts obtained by MAE.

Sample	RT (min)	[m-H]^+^, *	MW ^&^	Formula	LC-MS Experimental Identification	Tentative Aglycone Assignment	Database Identifier (PubChem CID)
1	0.76	474.22	474	C_9_H_8_O_3_	p-Coumaroyl tartaric acid glucosidic ester	p-Coumaric acid	PubChem: 637542
2	14.76	518.32	518.424	C_24_H_22_O_13_	6″-O-Malonylgenistin	Malonylgenistin	PubChem: 15934091
3	15.29	611.42	611.525	C_27_H_31_O_16_	Cyanidin 3,5-O-diglucoside	Cyanidin	PubChem: 441688
4	16.58	546.40	544.504	C_27_H_28_O_12_	3,4-Diferuloylquinic acid	Diferuloylquinic acid	PubChem: 102036608
5	18.57	558.46	558.617	C_30_H_38_O_10_	Secoisolariciresinol-sesquilignan	Secoisolariciresinol-sesquilignan	PubChem: 59728532
6	24.29	654.48	652.554	C_29_H_32_O_17_	Quercetin 3-O-(6″-acetyl-galactoside) 7-O-rhamnoside	Quercetin	PubChem: 14353459

* Experimental data. ^&^ Theoretical data. Note: Glycosylated or derivatized compounds were simplified to their corresponding aglycone forms for clarity. Compound identification is considered putative based on LC-MS/MS data.

**Table 3:** Putative compound identification of extracts obtained by UAE.

Sample	RT (min)	[m-H]^+^, *	MW ^&^	Formula	LC-MS Experimental Identification	Tentative Aglycone Assignment	Database Identifier (PubChem CID)
1	0.42	223.98	224.21	C_11_H_12_O_5_	Sinapic acid	Sinapic acid	PubChem: 10743
2	0.77	474.22	474	C_9_H_8_O_3_	p-Coumaroyl tartaric acid glucosidic ester	p-Coumaric acid	PubChem: 637542
3	2.38	611.16	611.527	C_30_H_27_O_14_	Cyanidin 3,5-O-diglucoside	Cyanidin	PubChem: 441688
4	11.86	460.49	462.36	C_21_H_18_O_12_	Kaempferol 3-O-glucuronide	Kaempferol	PubChem: 5318759
5	16.24	678.48	680.564	C_30_H_32_O_18_	Chrysoeriol 7-O-(6″-malonyl-apiosyl-glucoside)	Chrysoeriol	PubChem: 157009732
6	17.3	705.53	706.646	C_36_H_34_O_15_	(-)-Epicatechin-(2a-7)(4a-8)-epicatechin 3-O-galactoside	Epicatechin	PubChem: 157009728
7	23.09	636.46	638.484	C_27_H_26_O_18_	Luteolin 7-O-diglucuronide	Luteolin	PubChem: 157009729

* Experimental data. ^&^ Theoretical data. Note: Glycosylated or derivatized compounds were simplified to their corresponding aglycone forms for clarity. Compound identification is considered putative based on LC-MS/MS data.

## Data Availability

The data presented in this article cannot easily be made public due to confidentiality agreements. Requests to access the datasets should be directed to the first or last author.

## Abbreviations

The following abbreviations are used in this manuscript:

MAE	Microwave-assisted extraction
UAE	Ultrasound-assisted extraction
PSF	Pomegranate seed flour
TPC	Total phenolic content
TFC	Total flavonoid content
